# Supplementary material for: Mint/X11 PDZ domains from non-bilaterian animals recognize and bind CaV2 calcium channel C-termini in vitro
Source: Sci Rep. 2024 Sep 16;14:21615. doi: 10.1038/s41598-024-70652-8 (PMC11405698; doi:10.1038/s41598-024-70652-8)
Supplement: Supplementary file 18 — Supplementary Information 18. [file 41598_2024_70652_MOESM18_ESM.pdf]

Supplementary File 17. Raw images of Western blots shown in figures 6G and S3C.

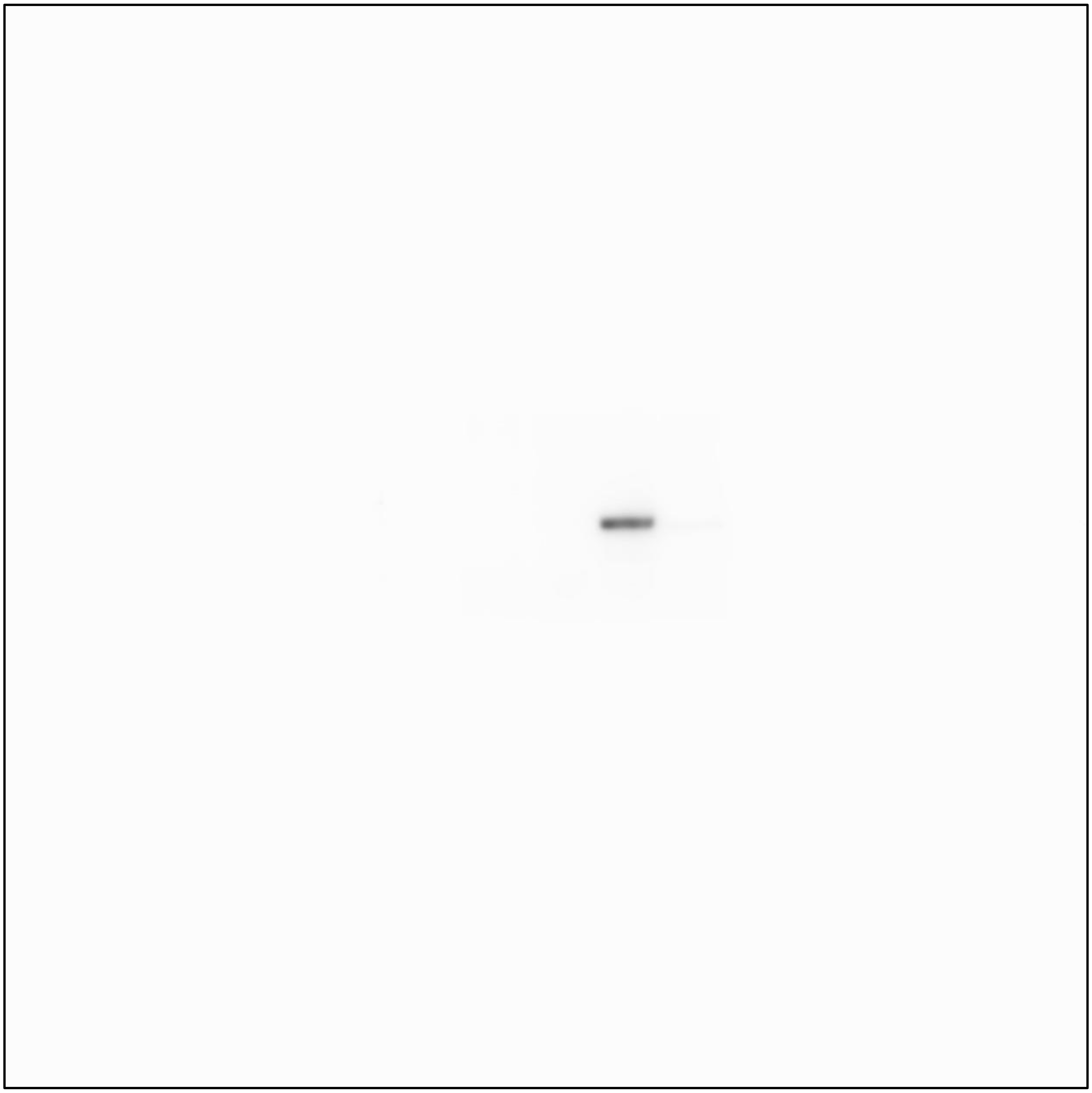

Top panel, figure 6G.

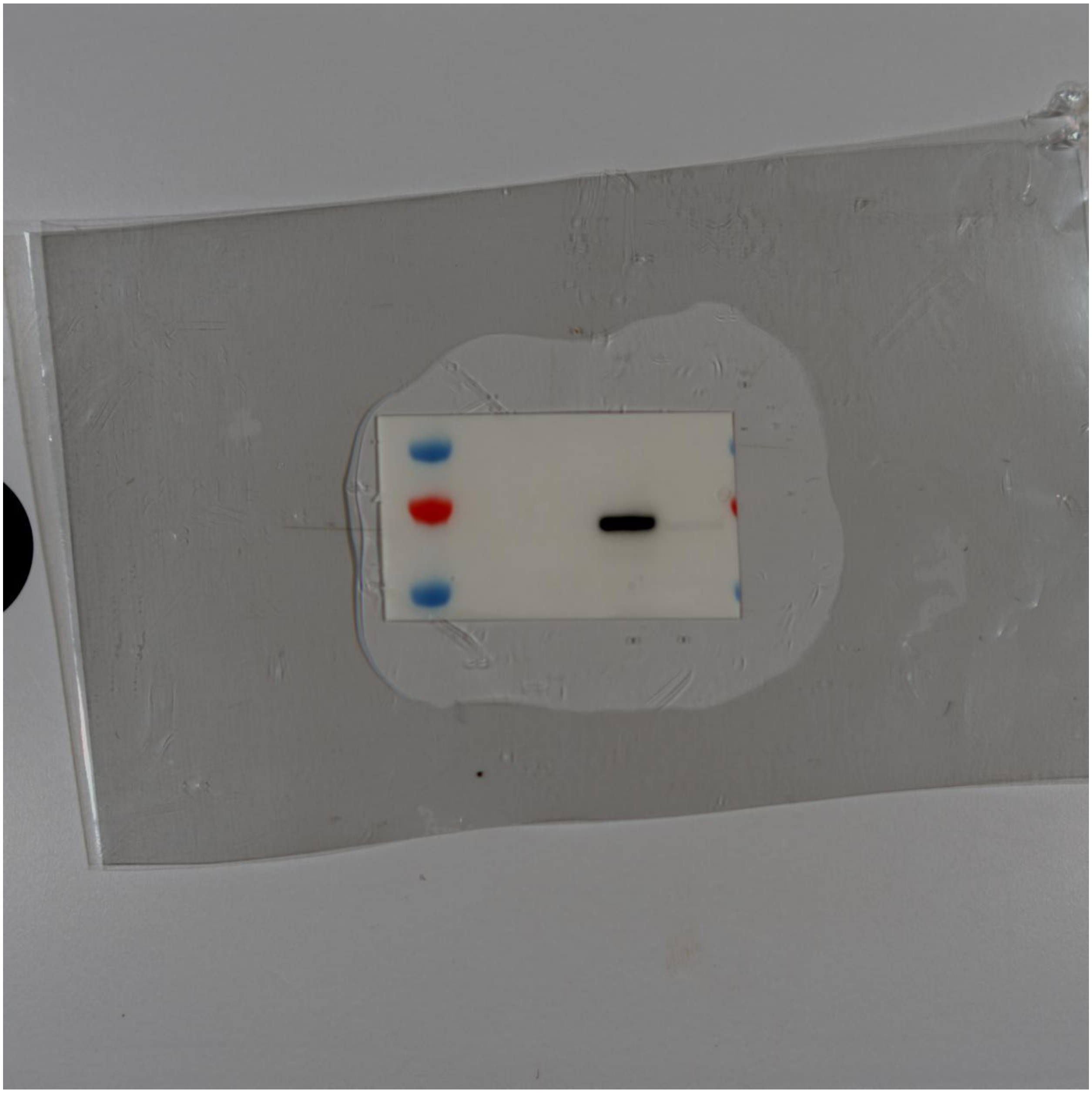

Alternate image, top panel, figure 6G.

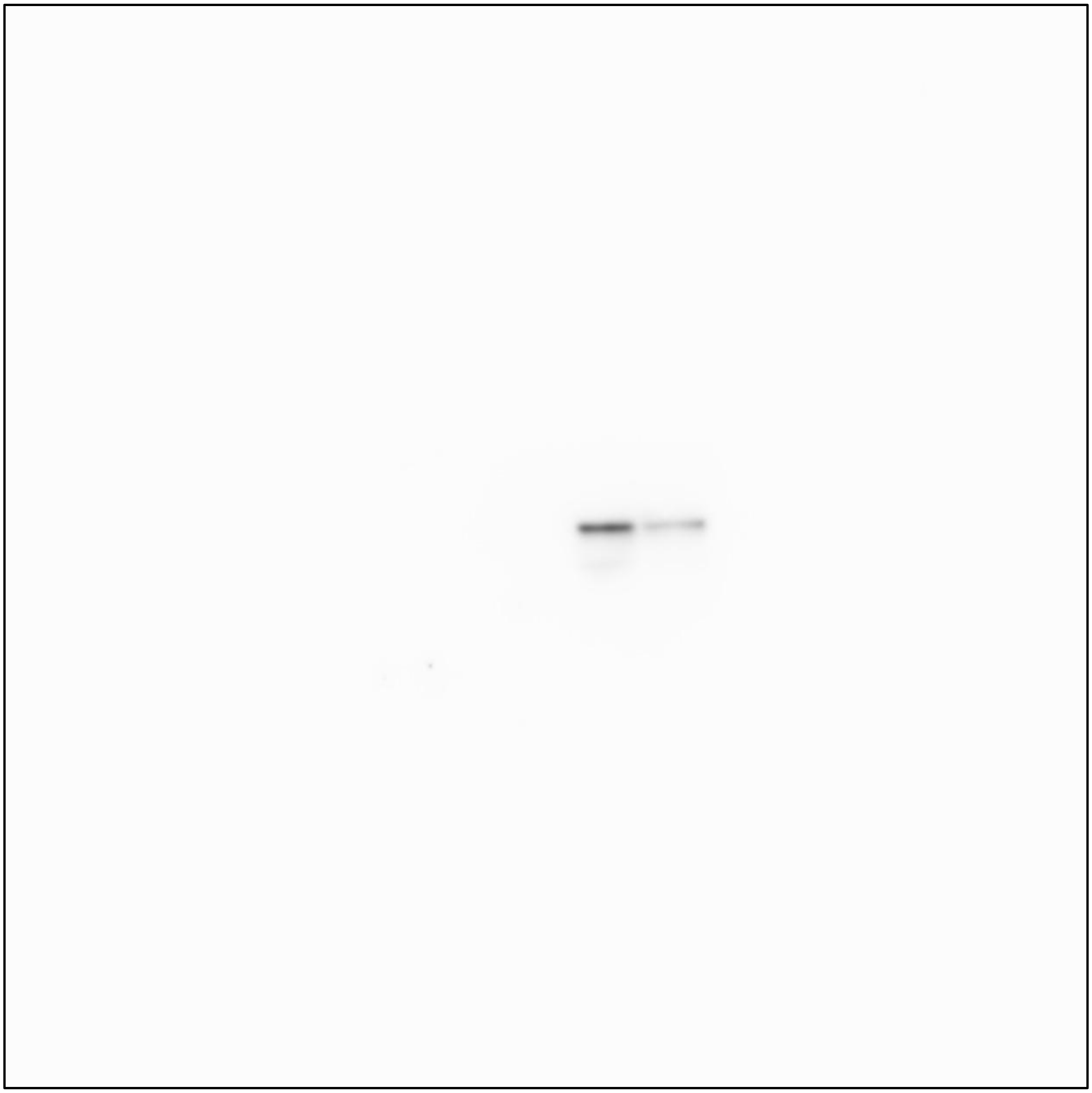

Top-middle panel, figure 6G.

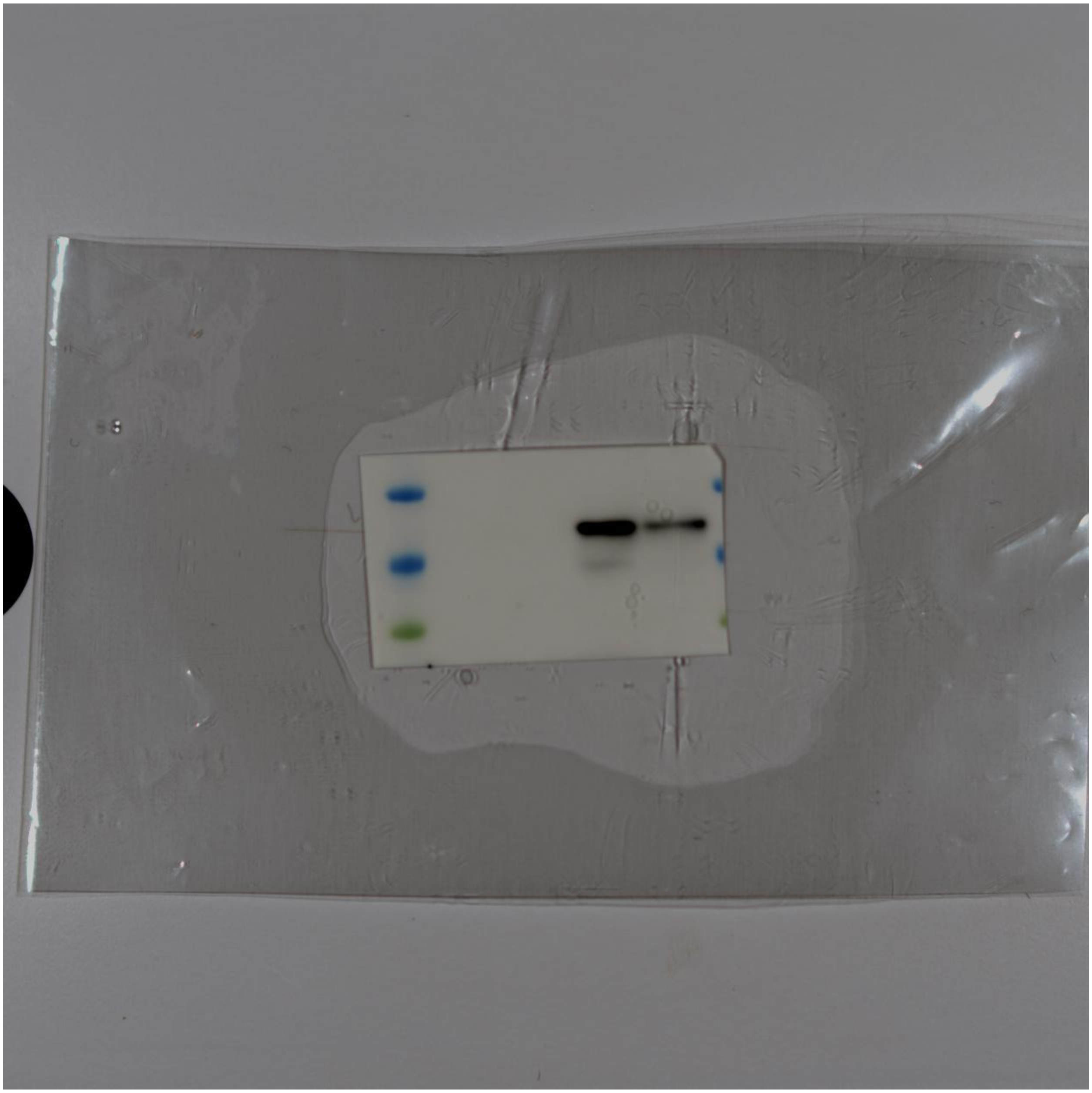

Alternate image, top-middle panel, figure 6G.

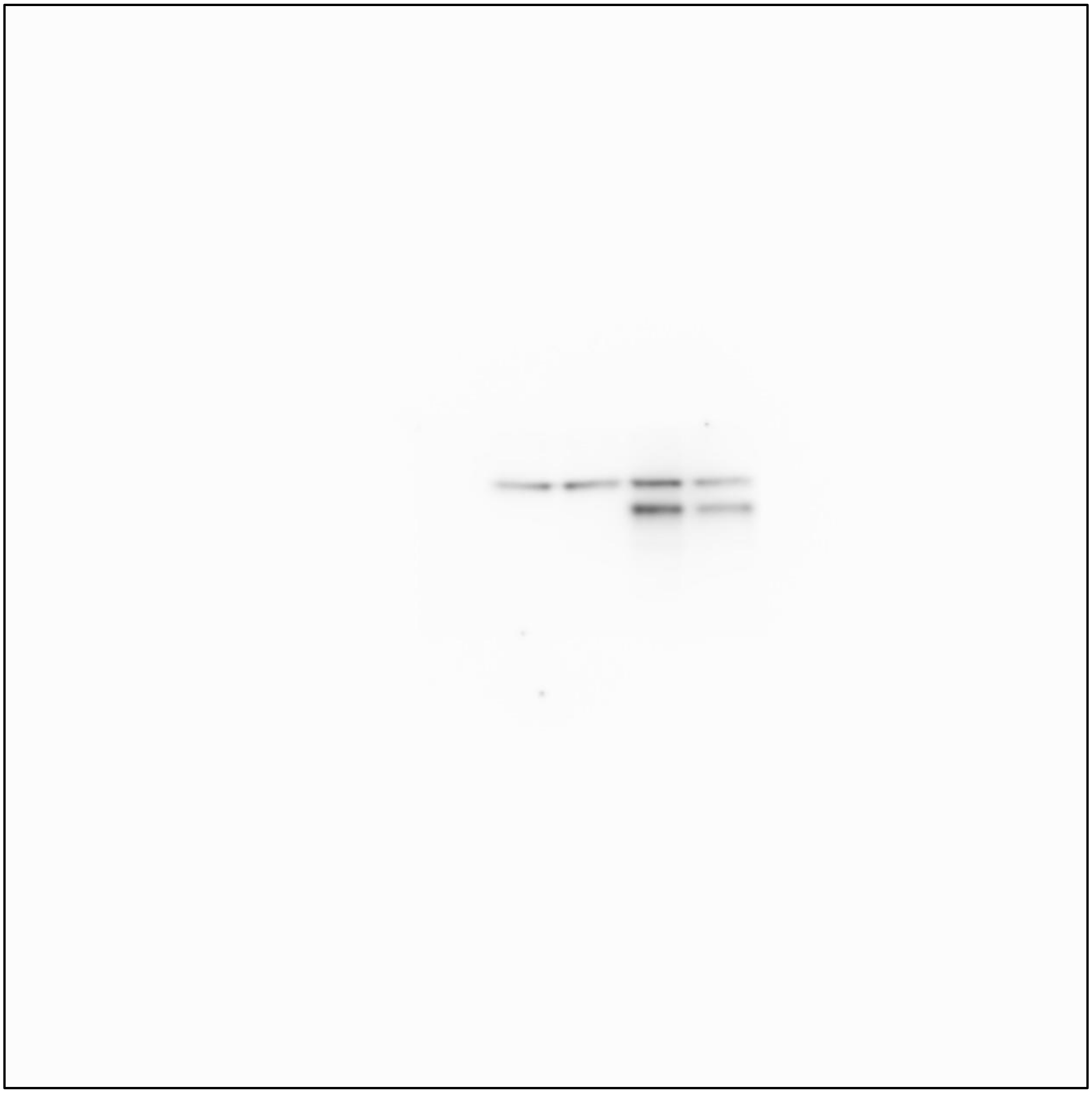

Bottom-middle panel, figure 6G.

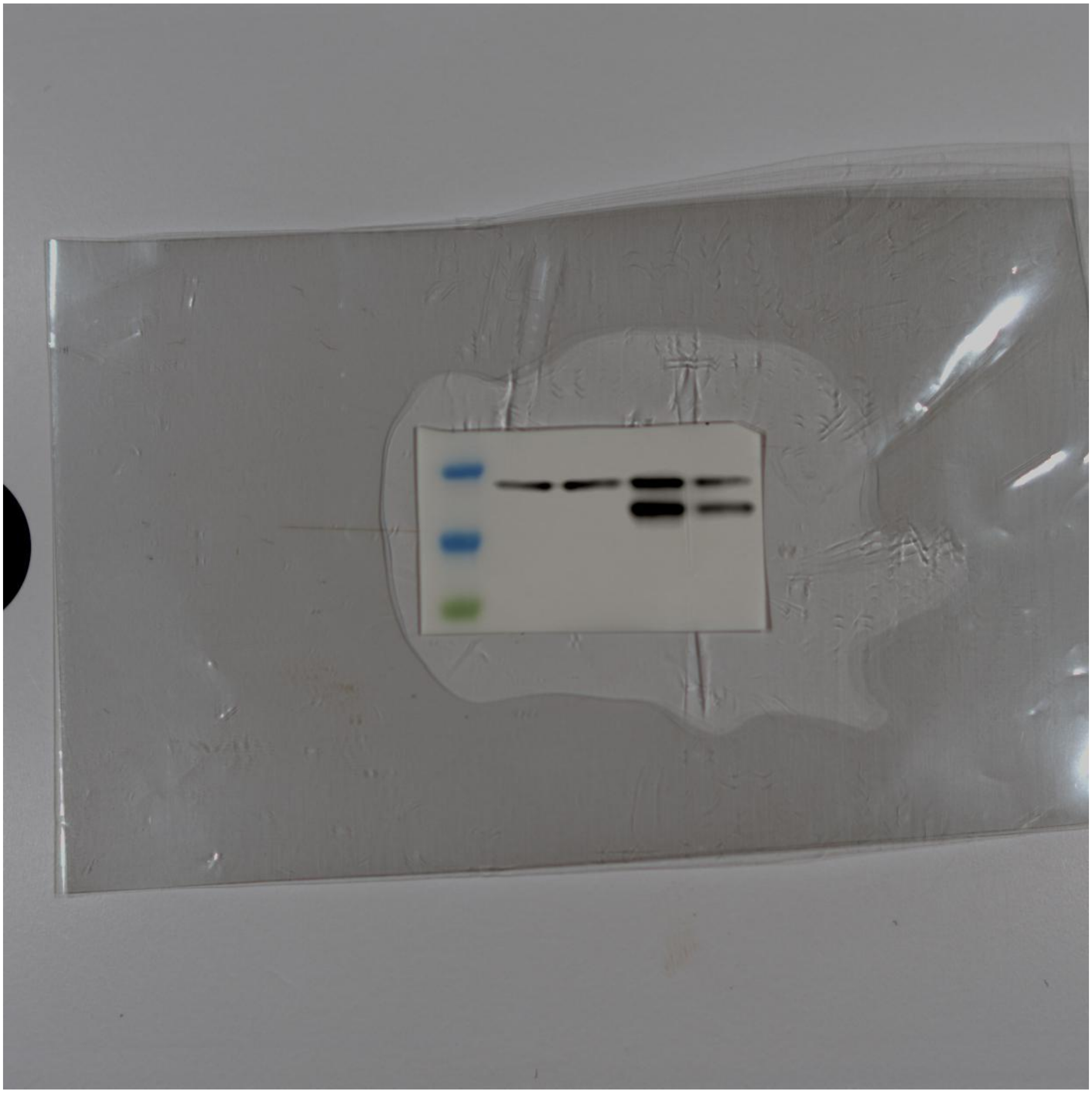

Alternate image, bottom-middle panel, figure 6G.

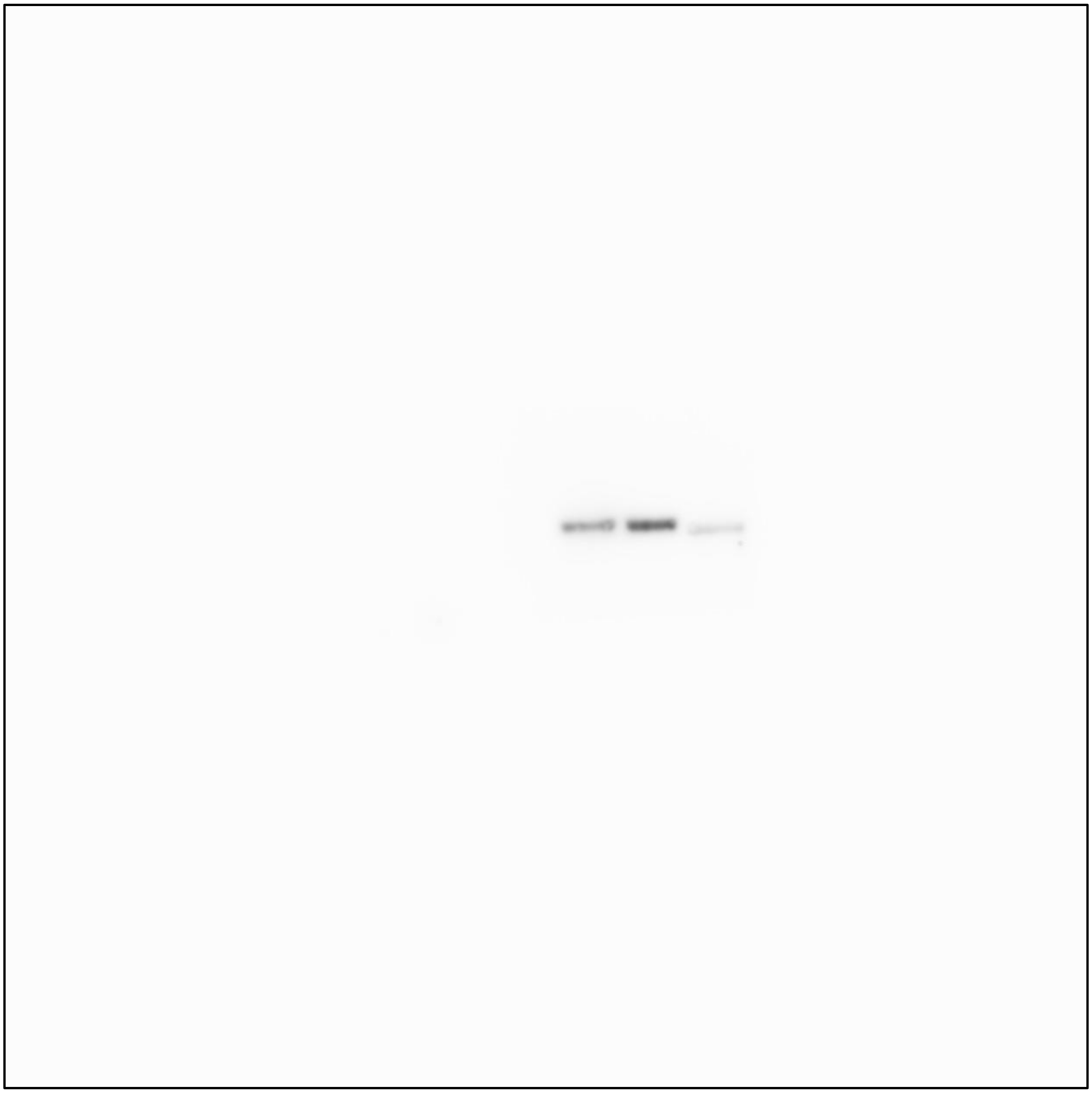

Bottom panel, figure 6G.

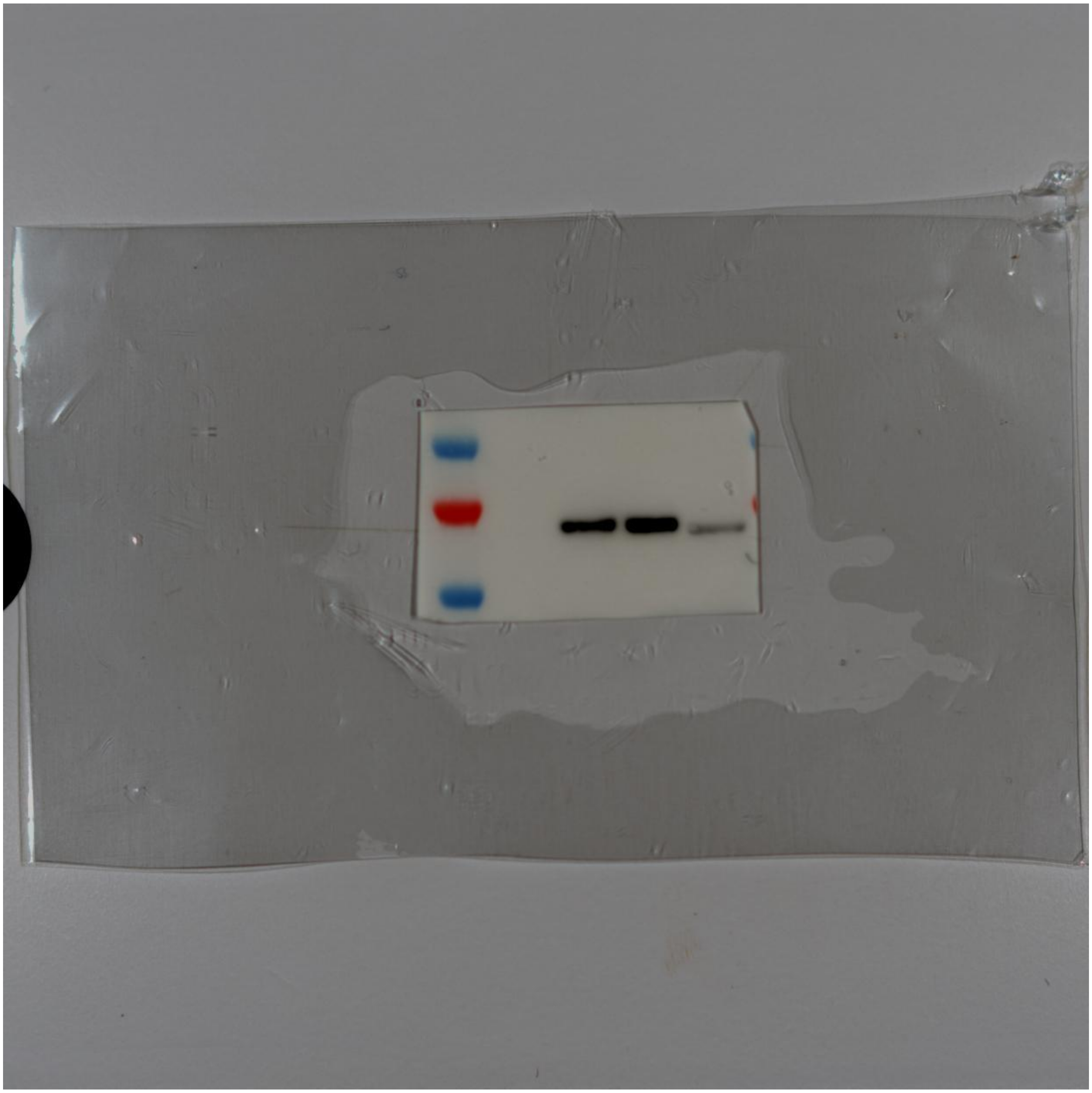

Alternate image, bottom panel, figure 6G.

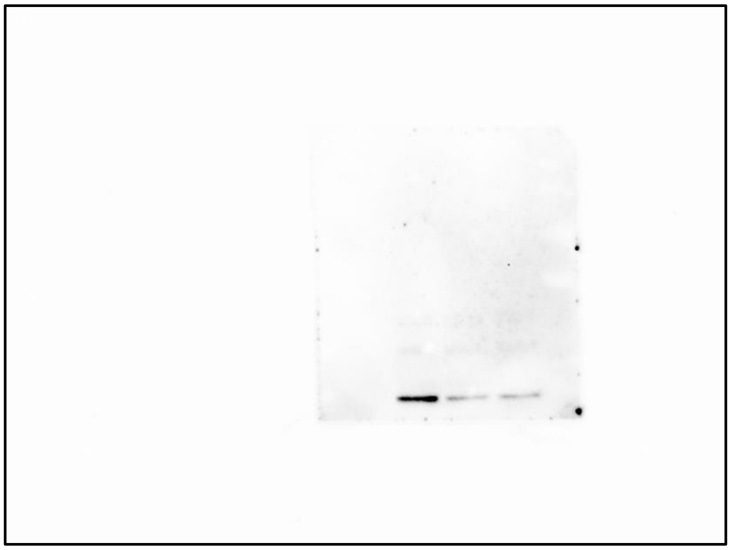

Top panel, figure S3C.

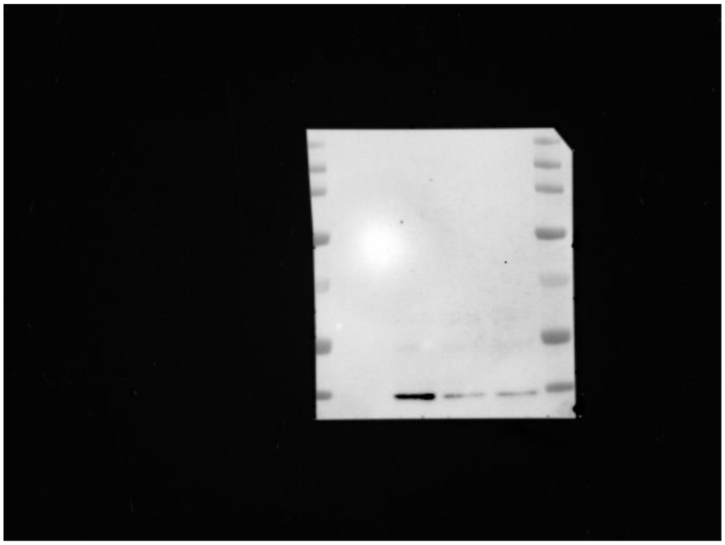

Alternate image, top panel, figure S3C.

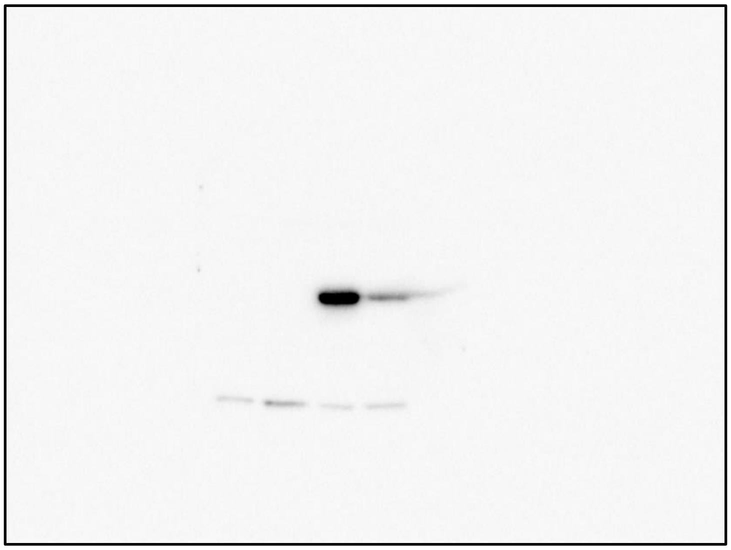

Top-middle panel, figure S3C.

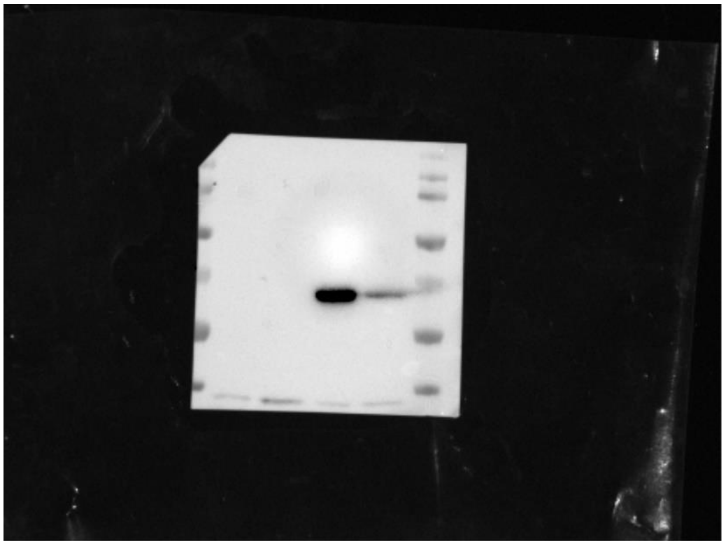

Alternate image, top-middle panel, figure S3C.

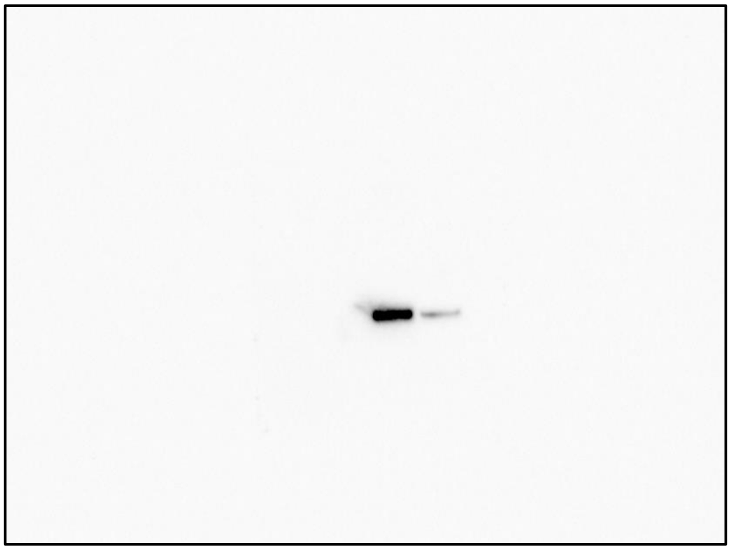

Bottom-middle panel, figure S3C.

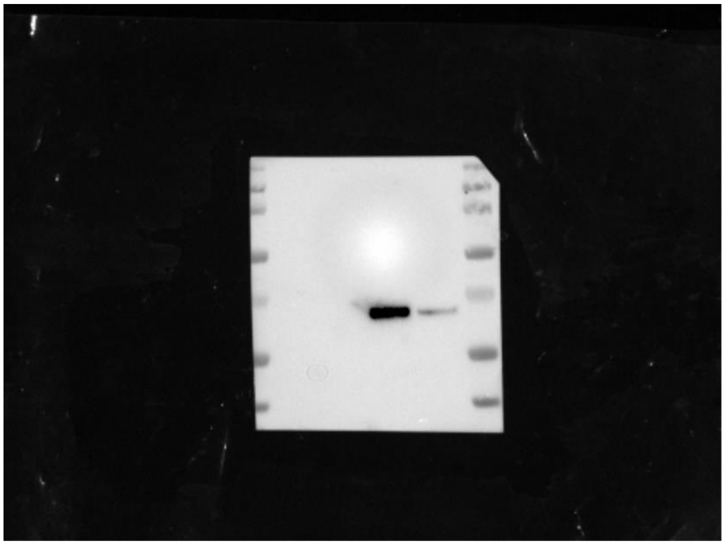

Alternate image, bottom-middle panel, figure S3C.

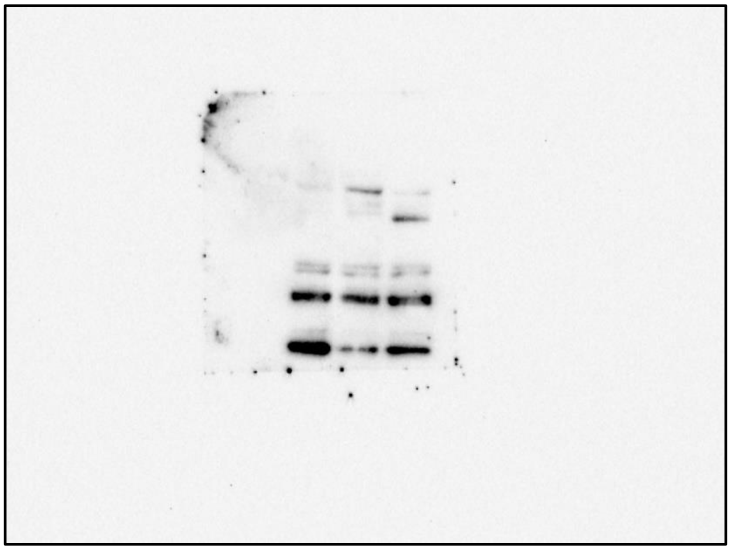

Bottom panel, figure S3C.

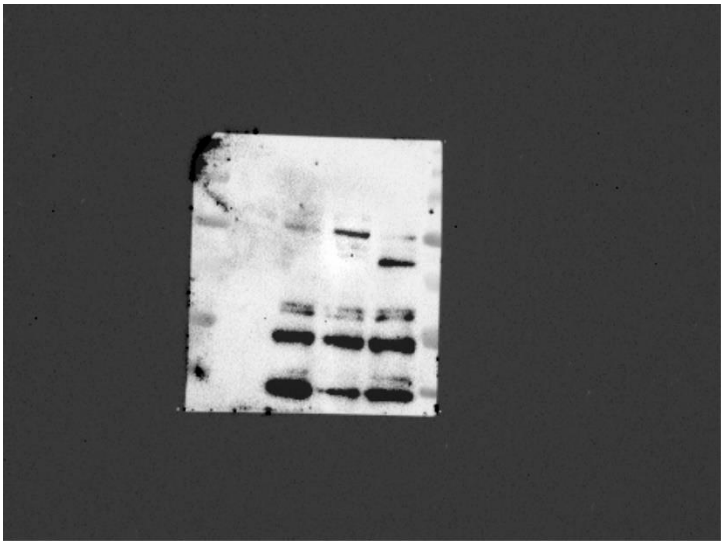

Alternate image, bottom panel, figure S3C.
